# Impact of Motivation on Selected Aspects of Attention in Children with ADHD

**DOI:** 10.1007/s10578-020-01042-0

**Published:** 2020-08-20

**Authors:** Sebastian Skalski, Grzegorz Pochwatko, Robert Balas

**Affiliations:** Institute of Psychology, Polish Academy of Sciences, 1 Jaracza Street, 00-378 Warsaw, Poland

**Keywords:** ADHD, Vigilance, Visual search, Divided attention, Motivation

## Abstract

Earlier reports showed the co-occurrence of a motivation deficit in children with ADHD. The purpose of this study was to assess the impact of extrinsic motivation on selected aspects of attention in children with ADHD, as well as to measure cortical activity and dimensions of motivation as per the self-determination theory. The study included 30 children with ADHD and 30 typically developing (TD) children aged 9–13 years. Children with ADHD exhibited a higher theta/beta power ratio (TBR) in the midline and a lower regional cerebral blood oxygenation (rCBO_2_) level in prefrontal areas measured using the HEG ratio compared to TD children. Children with ADHD were more likely to undertake activity under the pressure of external stimuli and exhibited attention deficits regarding vigilance, visual search and divided attention. Differences between groups regarding attention decreased in conditions of increased motivation, indicating that motivation can reduce cognitive deficits in children with ADHD.

## Introduction

Attention Deficit Hyperactivity Disorder (ADHD) is the most common neurobehavioral childhood disorder. Its incidence is estimated at 3–8% in the age population of < 18 [1–3]. ADHD is characterized by symptoms of impulsivity, hyperactivity and inattentive behavior which are incommensurate with a patient’s age [4]. ADHD may cause problems in psychosocial functioning, such as deteriorated academic performance or problems in relations with family and peers. The symptoms are often accompanied by low self-esteem, depressive states, anxiety disorder and oppositional defiant disorder [5–8]. Three ADHD types of presentations can be distinguished: the predominantly hyperactive-impulsive type (ADHD-HI), the predominantly inattentive type (ADHD-I) and the combined type (ADHD-C) [9]. When analyzing the underlying mechanisms of ADHD, special role is attributed to abnormalities in the functioning of neuronal connections between the cortex (mainly frontal lobes) and basal nuclei [10]. This is also associated with abnormal neurotransmitter activity, mainly dopamine and noradrenaline [11, 12]. Moreover, children with ADHD show a 3–4% decrease in the total brain volume. However, this difference does not apply to the entire cerebral tissue, but primarily to the frontal lobe cortex, basal nuclei, cerebellum and the corpus callosum [13, 14]. Studies using diffusion tensor imaging (DTI) have revealed widespread abnormalities regarding brain white matter integrity in children with ADHD [15]. Studies of brain function in children with ADHD have shown a decrease in blood flow (hypoperfusion) in the frontal lobes and basal nuclei [16–18]. Other studies have shown increased theta activity in the frontal areas, increased delta activity and decreased alpha and beta activity in the occipital areas [19–21]. EEG (electroencephalography) recording also revealed the occurrence of numerous epileptiform grafoelements [22]. Quantitative EEG (QEEG) analysis is used to search for neuromarkers in the diagnosis of ADHD, especially the analysis of the theta/beta power ratio (TBR) (high in children with ADHD) in central areas [23, 24]. According to Snyder et al. [25], TBR analysis can improve the diagnostic accuracy of attention deficit disorder by more than 25% compared to the use of clinical interviews alone. However, the literature data are not unanimous—not all studies have confirmed significant (large) TBR differences between children with ADHD and typically developing (TD) children [26, 27]. According to recent studies, significant differences between the groups can also be observed using hemoencephalography (HEG) in the prefrontal regions [28, 29], yet the number of reports in this respect is limited. Further research is needed to better understand the neuronal ADHD correlates. Moreover, the determination of ADHD neuromarkers seems to be crucial in the diagnosis of ADHD because it is an objective indicator of ADHD, which may precede the detection of ADHD using classical methods. The use of EEG and HEG recordings for this purpose seems reasonable, as these types of devices can be widely used—they are inexpensive and low-maintenance, easy to operate, and do not require additional space in the clinic or laboratory.

Studies using functional magnetic resonance imaging (fMRI) have shown that reduced activation in the prefrontal cortex is associated with attention deficit in children with ADHD [30]. Attention is considered the mechanism (system) responsible for selecting information and countering the undesirable effects of overloading the cognitive system [31]. Its aspects include selective (focused) attention, visual search (scanning), vigilance, divided attention and alternating attention. Selective attention enables focusing on selected stimuli and rejecting disruptive or irrelevant information [32]. Visual search allows for an active and systematic search of the perceptive field in order to detect objects which fit an adopted criterion [33]. Vigilance enables long-term monitoring of the environment to detect a specific signal while disregarding distractors [34]. Divided attention is responsible for the ability to focus on two or more sources of information at the same time [35]. Meanwhile, alternating attention allows for relatively fast switching between two objects (tasks), which are carried out as part of independent information processing [36]. According to previous studies, children with ADHD may show impairment concerning vigilance, visual search and divided attention [37–42]. However, researchers reveal varying magnitude of effects, which may indicate neurocognitive heterogeneity in ADHD [43]. It also seems that cognitive deficits in children with ADHD are not permanent neuropsychological impairments. Extrinsic motivation (e.g. the prospect of receiving a reward) can increase the cognitive capacity in children with ADHD [44, 45]. A positive impact of motivation was observed with regard to visual search, working memory and inhibitory control. Studies on vigilance and divided attention have not yet been conducted. Furthermore, it is unclear whether motivation may completely eliminate attention deficits in children with ADHD.

Apart form neuronal ADHD correlates, researchers are trying to establish how behavior patterns of children with deficits are linked to motivation. Motivation helps to explain human behavior, its direction and duration [46]. Deci and Ryan [47, 48] differentiate 3 types of motivation: intrinsic motivation, extrinsic motivation and amotivation. The first one defines individual, spontaneous interests of individuals. The second one takes into account the consequences of human behavior and has an instrumental function. The last one allows for explaining the non-autonomous activity of individuals with no regulation. In the literature on the subject, amotivation is often compared to the concept of learned helplessness according to Seligman [49]. Proper extrinsic motivation is a continuum of internally and externally regulated states of varying intensity: external, introjected, identified, and integrated regulation. In the case of external regulation, behavior occurs only under the influence of environmental stimuli. Introjected regulation initiates activity due to possible sanctions and awards. Identified regulation allows for assigning personal meanings to the undertaken behavior. In the case of integrated regulation, the activity is tailored to the objectives of the individual and undertaken in accordance with one’s sense of self. The self-determination theory by Deci and Ryan allows for unifying the perception of human behavior based on commitment [50]. In the literature on the subject, an increasing number of researchers are observing the co-occurrence of motivation deficits in children with ADHD [44, 45, 51–53]. Children with ADHD require stronger stimuli to change their behavior, and have difficulty in postponing gratification. They prefer small and immediate reinforcement to larger and delayed reinforcement [54, 55]. Based on the described behavior samples, it seems that children with ADHD may exhibit a higher level of extrinsic motivation, introjected motivation and amotivation, as well as a lower level of intrinsic motivation and identified regulation; however, there are no studies describing the activity of children with ADHD as per the self-determination theory.

### Objective of the Study

The purpose of this study was to assess the impact of extrinsic motivation on selected aspects of attention in children with ADHD, as well as to measure cortical activity and dimensions of motivation as per the self-determination theory.

Based on the aforementioned articles, we assumed that: (H1) children with ADHD may exhibit lower levels of cortical activity as measured using (a) TBR in the midline and the (b) HEG ratio in prefrontal areas than TD children; (H2) children with ADHD may display lower levels of (a) intrinsic motivation and (b) identified regulation as well as higher levels of (c) external regulation, (d) introjected regulation and (e) amotivation to learning compared to TD children; (H3) children with ADHD may exhibit deficits regarding: (a) vigilance, (b) visual search and (c) divided attention; (H4) increasing the motivation may reduce attention deficits in children with ADHD with regard to: (a) vigilance, (b) visual search and (c) divided attention. The obtained data will enable a better understanding of ADHD issues.

## Methods

The study was conducted in the fall of 2019. The participation in the study was voluntary. Consent was required of the patient and their parent or legal guardian. This study was approved by The Committee for Ethics in Scientific Research of The Institute of Psychology, Polish Academy of Science (Research project approval # 15/VIII/2019).

### Procedure

The measurement of cortical activity, fluid intelligence and motivation to learning was conducted at the beginning of the study. The assessment of the impact of motivation on selected aspects of attention was carried out in a quasi-experiment model with repeated measurement. The manipulative technique consisted in increasing the motivation in all of the examined participants. For a correct result as part of each of the tasks (gain), participants received a sweet reward (chocolate). In addition, a named record board for the top 10 results was introduced—competition effect. The manipulative technique corresponds with previous studies by McInerney and Kerns [44], as well as Reijnen and Opwis [45] on the impact of motivation on cognitive function in children with ADHD.

### Participants

The study involved 30 children (24 boys and 6 girls) aged 9–13 years, diagnosed with ADHD by a specialist in pediatric psychiatry or neurology. Moreover, the diagnosis was confirmed by a structured diagnostic interview for psychomotor hyperactivity as per DSM-V [56], conducted by a psychologist. Eight (8) children remained in pharmacotherapy (methylphenidate), but did not take drugs 48 h prior to the examination—at the doctor’s consent. The comparison group consisted of 30 TD children (23 boys and 7 girls) aged 9–13. The recruitment conditions for both groups included lack of neurological diseases as well as intellectual capacity within standard. Recruitment was conducted among patients of psychological and pedagogical support centers in Kraków (Poland).

### Measures

As part of the study, cortical activity was measured using:

**TBR** EEG was recorded using a 10-channel FlexComp Infiniti encoder (up to 2048 samples/s) with impedance control (less than 5 kΩ). Two additional reference electrodes were placed on the earlobes. Recording was conducted in resting state with eyes open for 3 min. Data were processed in Biograph Infiniti 6.2. The signal was filtered in the 0.50–30 Hz band. Artifacts were corrected by automatic elimination. Amplitudes above 70 µV were removed along with the artifacts. The results were subjected to the Fast Fourier Transform (FFT) analysis. TBR was calculated from the ratio of theta power (4–7 Hz waves) to beta power (13–30 Hz waves) based on the mean value registered using electrodes placed in central areas: C3, Cz, C4, according to the 10:20 placement.

**HEG Ratio** The HEG system (32 samples/s) using near infrared spectroscopy (NIRS) was employed for recording regional cerebral blood oxygenation (rCBO_2_). The measurement method uses different optical properties of hemoglobin (Hb) and oxyhemoglobin (oxy-Hb). It was described in detail by Toomim et al. [57]. Reflected light penetrates to a depth of 1.5 cm and reaches the capillaries in the gray matter at the base of the cerebral cortex. Recording was conducted in resting state with eyes open for one minute. Data were processed in Biograph Infiniti 6.2. The HEG ratio was calculated using the following formula: HEG Red/HEG IR × 200, where HEG Red denotes the values of reflected red light (660 nm), while HEG IR denotes the values of reflected infrared light (850 nm) based on the mean value recorded using optodes placed in prefrontal areas: Fp1, Fpz, Fp2, according to the 10:20 placement.

Moreover, the study used psychological tools to determine the level of intelligence, motivation and attention:

**Raven’s colored progressive matrices (CPM)** in Polish standardization [58] for measuring educational capacity (fluid intelligence) in children. CPM contains 36 tasks in the form of incomplete patterns (matrices). The task of the examined person is to select the missing fragment from among the given proposals.

**Situational motivation scale (SMS-15)** [59] for measuring motivation based on the self-determination theory [47, 48]. The questionnaire consists of 15 statements grouped under 5 factors: intrinsic motivation (Chronbach’s α = 0.82), external regulation (α = 0.82), introjected regulation (α = 0.84), identified regulation (α = 0.84) and amotivation (α = 0.82). The test taker expresses their attitude towards each of the statements on a 7-point Likert scale, where 1—“I strongly disagree” and 7—“I strongly agree”.

**Shortened version of the Mackworth clock task** [60] for measuring vigilance. The examined person observes a clock hand moving on the screen. The hand moves in short jumps every 1 s, like the second hand of an analog clock. At rare and irregular intervals, the hand makes a double jump by two seconds. The task lasts 5 min (300 hand jumps). During the test, there are 18 irregular hand jumps (6% probability). The task of the examined person is to detect the double (irregular) jumps by pressing a button. The number of omission and commission errors is recorded.

**Visual search task** for assessing a conjunctive search, i.e. with a specific combination of two stimulus characteristics (color and angle of rotation). The role of the examined person is to scan the perception field in order to detect (by pressing a button) the red T letter among inverted red T letters and blue T letters. The probability of occurrence of an element complying with the criterion is 40%. The task consists of 48 boards with 5–20 elements on the screen. Recording takes place of the average reaction time (RT) and RT slope, which reflects the average RT increase at the moment of each additional element appearing on the screen.

**Multitasking test** for measuring divided attention. The role of the examined person is to observe a screen divided into 2 parts and to react according to the location of the stimulus. If a figure appears in the upper part of the screen, the test taker should react (the appropriate button) according to the shape of the figure (square or rhombus), and if the figure is placed in the lower part, they should react accordingly to the number of dots in the middle of the figure (two or three dots). The task consists of 48 items. 24 boards for single-tasking (reaction according to one stimulus feature) and 24 boards for multi-tasking (reaction according to two stimulus features interlaced with one another).

The tests we selected are popular among neuropsychologists and are often used to assess brain performance. The measurement of selected attention aspects was carried out using computer applications in the Java environment. The tasks were presented on a 19-inch screen. The distance of the examined person from the screen was about 70 cm. Each test was preceded by a short training session.

### Data Analysis

Statistical data analysis was conducted in IBM SPSS Statistics 26. The normality distribution was verified using the Kolgomorov–Smirnov test. Levene’s test was used to assess the homogeneity of variance. In most cases, the obtained results allowed for the use of parametric tests (except for the participants’ age analysis). In order to determine the significance of differences, the Student’s t-test, Mann–Whitney’s U test and the repeated measures multivariate analysis of variance ANOVA were used. The repeated measures ANOVA did not include the sphericity test due to two levels of independent variable within each effect. The level of significance was set at *p* < 0.05. The effect size was assessed on the basis of a partial eta square (*η*²) or the *r* coefficient.

## Results

The study did not contain statistically significant differences between children with ADHD and TD children in terms of age and intellectual capacity measured using CPM (each participant scored above the 50th centile).

The Student’s t-test confirmed the occurrence of statistically significant differences between children with ADHD and TD children regarding: TBR, HEG ratio and motivation dimensions. Detailed analysis results are presented in Table 1.

**Table 1:** Differences in mean values: age of participants, intellectual capacity, cortical activity, dimensions of motivation in children with ADHD and TD children

	ADHD children (N = 30)		TD children (N = 30)		U/t	r
	M	SD	M	SD		
Age	10.83	1.68	10.67	1.35	429	0.06
CPM	69.53	13.28	71.77	9.28	− 0.76	− 0.10
TBR (theta/beta power ratio)						
C3	4.29	1.09	3.11	0.80	4.77***	0.53
Cz	4.66	1.36	3.64	0.88	3.45**	0.41
C4	4.12	1.09	2.93	0.72	4.98***	0.55
HEG ratio						
Fp1	76.03	7.42	98.81	14.78	− 7.54***	− 0.70
Fpz	81.38	8.82	102.91	10.65	− 8.53***	− 0.75
Fp2	77.78	8.71	100.74	13.05	− 8.02***	− 0.73
Motivation						
INM	10.07	4.21	14.43	3.80	− 4.22***	− 0.48
EXR	17.03	2.88	12.93	2.79	5.61***	0.59
INTR	16.30	3.98	13.63	3.40	2.79**	0.34
IDE	15.07	3.62	17.73	3.31	− 2.97**	− 0.36
AMO	14.17	4.24	11.83	3.44	2.34*	0.29

*ADHD* attention deficit hyperactivity disorder, *TD children* typically developing children, *CPM* Raven’s colored progressive matrices (result in centiles), *INM* intrinsic motivation, *EXR* external regulation, *INTR* introjected regulation, *IDE* identified regulation, *AMO* amotivation, *M* mean, *SD* standard deviation, *U/t* Mann–Whitney’s U-test result for age (no normal distribution), Student’s t-test results for the other variables, *r* effect size

Significance levels: *p < 0.05; **p < 0.01; ***p < 0.001

Next, a mixed design two-way MANOVA was conducted: 2 (group: *children with ADHD* versus *TD children*) × 2 *(normal conditions* versus *increased motivation*). The factor measured between individuals was membership to one of the research groups, whereas motivation was the factor measured internally. Dependent variables included selected aspects of attention. As a result of the analyses, a multidimensional interaction effect was obtained regarding group membership and motivation, *F*(6,53) = 8.13, *p* < 0.001, *η*² = 0.48. Due to the formulated research questions, main effects were not analyzed. Univariate *F* tests confirmed the interaction effects for the following dependent variables: omission errors, *F*(1,58) = 19.58, *p* < 0.001, *η*² = 0.25; RT in visual search of 5–20 elements, *F*(1,58) = 17.59, *p* = 0.001, *η*² = 0.23; RT slope in visual search, *F*(1,58) = 7.84, *p* = 0.007, *η*² = 0.12; RT in single-tasking, *F*(1,58) = 18.76, *p* < 0.001, *η*² = 0.24; as well as RT in multi-tasking, *F*(1,58) = 7.94, *p* = 0.007, *η*² = 0.12. No significant interaction effect was obtained for the commission errors dependent variable, *F*(1,58) = 0.09, *p* = 0.760, *η*² < 0.01.

In order to explain what the interaction effects involve, an analysis of simple main effects was conducted (the compared mean values are presented in Table 2).

**Table 2:** Mean scores obtained in the interaction between the group and motivation factors

	ADHD children (N = 30)				TD children (N = 30)			
	Normal conditions		Increased motivation		Normal conditions		Increased motivation	
	M	SD	M	SD	M	SD	M	SD
Vigilance								
Omission errors	8.87	2.36	7.13	2.58	6.17	3.09	5.90	2.80
Commission errors	6.70	2.67	6.63	2.66	5.03	2.16	4.93	2.16
Visual search (5–20 items)								
RT	4088.20	880.54	3192.70	757.78	3107.01	581.34	2927.80	443.49
RT slope	38.21	10.29	29.32	10.08	29.29	8.39	28.37	8.91
Multitasking RT								
Single-tasking	2845.17	751.50	2317.97	737.29	2221.47	663.64	2195.83	671.33
Multi-tasking	4445.03	1009.58	3908.07	998.33	3417.60	795.50	3395.13	781.56

*RT* reaction time in ms. Key to other elements: see Table 1

The group membership factor differentiated all of the controlled attention indicators under normal conditions: omission errors (*t* = 3.81, *p* < 0.001, *r* = 0.45), commission errors (*t* = 2.66, *p* = 0.010, *r* = 0.33), RT in visual search of 5–20 elements (*t* = 5.09, *p* < 0.001, *r* = 0.56), RT slope in visual search (*t* = 3.68, *p* = 0.001, *r* = 0.44), RT in single-tasking (*t =* 3.41, *p* = 0.001, *r* = 0.41) and RT in multi-tasking (*t* = 4.38, *p* < 0.001, *r* = 0.50).

The group membership factor differentiated the indicators of selected attention aspects in conditions of increased motivation: commission errors (*t* = 2.72, *p* = 0.009, *r* = 0.34) and RT in multi-tasking (*t* = 2.22, *p* = 0.031, *r* = 0.28). No significant differences were observed between ADHD and TD children in conditions of increased motivation for the following dependent variables: omission errors (*t* = 1.78, *p* = 0.081, *r* = 0.23), RT in visual search of 5–20 elements (*t* = 1.65, *p* = 0.105, *r* = 0.21), RT slope in visual search (*t* = 0.38, *p* = 0.702, *r* = 0.05) and RT in single-tasking (*t* = 0.67, *p* = 0.505, *r* = 0.09).

The motivation factor significantly differentiated the indicators of selected attention aspects in children with ADHD: omission errors (*t* = 4.12, *p* < 0.001, *r* = 0.48), RT in visual search of 5–20 elements (*t* = 3.95, *p* < 0.001, *r* = 0.46), RT slope in visual search (*t* = 3.10, *p* = 0.004, *r* = 0.38), RT in single-tasking (*t* = 4.49, *p* < 0.001, *r* = 0.51) and RT in multi-tasking (*t* = 4.33, *p* < 0.001, *r* = 0.49). In the case of commission errors, no significant differences in normal and increased motivation conditions were observed *(t* = 0.81, *p* = 0.423, *r* = 0.11).

In the case of TD children, all of the controlled attention indicators were similar in normal and increased motivation conditions: omission errors (*t* = 1.61, *p* = 0.118, *r* = 0.21), commission errors (*t* = 0.19, *p* = 0.854, *r* = 0.02), RT in visual search of 5–20 elements (*t* = 1.50, *p* = 0.145, *r* = 0.19), RT slope in visual search (*t* = 0.46, *p* = 0.651, *r* = 0.06), RT in single-tasking (*t* = 0.51, *p* = 0.613, *r* = 0.07) and RT in multi-tasking (t = 0.15, *p* = 0.886, *r* = 0.02).

**Fig. 1:**
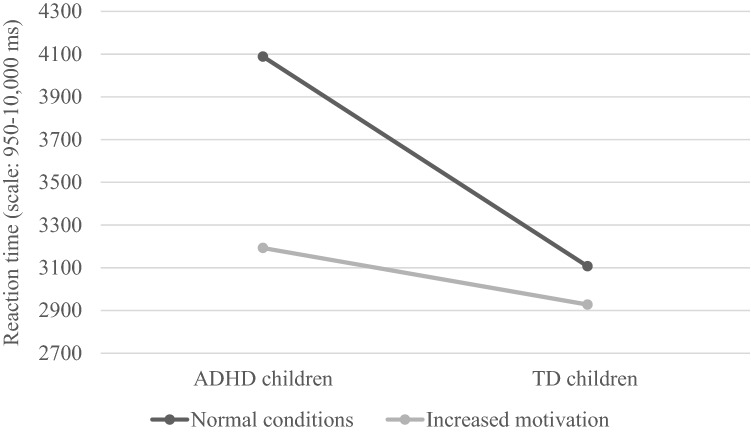
Differences in visual search of 5–20 elements on screen between ADHD and TD children in normal and increased motivation conditions (reaction time in ms)

In order to illustrate the interaction effect of group membership and motivation, Fig. 1 shows a chart of mean values in ANOVA using the example of the time of the visual search of 5–20 elements on screen.

## Discussion

Elevated TBR in children with ADHD has been well studied and described in the literature and has proven to be an effective aid in diagnosing attention deficits [6, 23, 24]. Our results are in line with the findings so far. Children with ADHD exhibited higher TBR in each of the controlled locations in the midline, i.e.: C3, Cz, C4 (Hypothesis 1a). The obtained effects should be considered medium or large (*r* ∈ 〈0.41, 0.55〉). In our study we did not correct individual alpha peaks. Some children with ADHD may display slow alpha peak frequencies instead of increased theta activity. Lack of this correction may overstate the TBR value. Langberg et al. [52] compared the TBR value in both conditions. Although less pronounced, these differences still remain significant. Moreover, it should be noted that most researchers reported greater effects for TBR measurement [61]. However, our study involved a non-homogeneous group of participants due to the ADHD type of presentation, while the EEG measurement itself was limited to 3 min. The majority of studies registered a 20-min recording, at different stages of cortical activity [62]. On the other hand, not all studies confirmed clear TBR differences between ADHD and TD children [26, 27].

HEG/NIRS is a method of visualizing cerebral perfusion and, consequently, brain activity. So far, few measurements have been performed using this technology. In our study, children with ADHD showed significantly lower levels of hemoglobin saturation with oxygen within each controlled location in the prefrontal areas, i.e.: Fp1, Fpz, Fp2 (Hypothesis 1b). The obtained data are in line with the limited literature in this respect [28, 29, 57]. Hypoperfusion in children with ADHD has also been confirmed by single-photon emission computed tomography (SPECT)—according to estimates, hypoperfusion affects 87% of children with ADHD [63]. Reduced blood flow in the prefrontal lobes may be associated with loss of inhibitory control, hyperactivity, impulsivity and inattentiveness, which is the clinical picture of ADHD [64, 65]. The obtained effects (*r* ∈ 〈0.70, 0.75〉) turned out to be larger than in the case of TBR differences.

Previous studies on motivation focused primarily on the lower achievement needs of children with ADHD and their biological conditioning, i.e. the limited availability of dopamine receptors in the reward system [53, 55]. In our study, we looked at the differences in the dimensions of motivation to learning between ADHD and TD children as per the self-determination theory (Hypothesis 2a–e). Children with ADHD were more likely to be active under pressure from external stimuli, for rewards or to avoid punishment (external and introjected regulation), and they also displayed a higher level of amotivation. TD children considered activity more self-determined (intrinsic motivation, identified regulation). The obtained results varied. The lowest one pertained to amotivation (*r* = 0.29), while the biggest one was noted in external regulation (*r* = 0.59). Our findings are consistent with the behavioral model of motivation in children with ADHD [55, 66].

Reports thus far have shown deficits in children with ADHD regarding vigilance [38, 41, 67]. Our study proved to support these findings (Hypothesis 3a). The obtained effects should be considered medium (*r* = 0.45 for omission errors, *r* = 0.33 for commission errors). Omission errors may indicate distraction, while commission errors may be a sign of impulsivity. It should be noted that some researchers reported greater difference effects compared to TD children [43]. However, our task lasted 5 min. In everyday conditions, the effects can be much greater, e.g. when long hours of vigilance at school is required or when cognitive stimuli (characteristic of the laboratory) are joined by sensory ones.

Children with ADHD displayed longer RT in the visual search task—large effect: *r* = 0.56, as well as a bigger RT slope—medium effect: *r* = 0.44 (Hypothesis 3b). Our results are consistent with many earlier studies using the conjunctive search method, which requires intensive selective visual attention [39, 40, 68]. Some studies also controlled the difficulty level of the task [39, 69, 70]. Interestingly, the largest effects were obtained at the highest, but also at the lowest level of difficulty. In the case of moderate complexity of a task, the differences between ADHD and TD children turned out to be small or insignificant. These data confirm the need to stimulate interest and use more frequent reinforcement in children with ADHD for tasks perceived as less demanding (motivational deficit), as well as to reduce requirements or divide complex (more difficult) tasks into smaller stages.

In the multitasking test for divided attention (Hypothesis 3c), children with ADHD showed longer RT in single-tasking (medium effect: *r* = 0.41) and in multi-tasking (large effect: *r* = 0.50), which is consistent with many previous reports in this respect [37, 42, 71]. The difference in response time for single-tasking confirms our findings to date about the slower rate of information gathering in children with ADHD. A bigger effect in multi-tasking indicates additional impairment of inhibitory control. It should be noted that, as per previous findings, the differences in multitasking are not due to deficits in prospective memory [42]. Children with ADHD have a similar ability to remember and recall test rules compared to TD children. In contrast, researchers observe a limited capacity to plan, organize behavior and monitor performance, which supports our findings on impairment of executive functions in children with ADHD [37, 42].

It should be noted that the identified deficits in selected aspects of attention in children with ADHD are not related to intellectual deficits, as the groups did not differ in their performance in this respect. We used only a non-verbal intelligence test, as all attention measurement tools were of a visual and spatial nature.

Our study confirmed the positive impact of extrinsic motivation on selected aspects of attention in children with ADHD, who have improved their capacity regarding most controlled aspects of attention. This impact did not apply to TD children who, in conditions of increased motivation, achieved the same results in normal conditions. Motivated, children with ADHD exhibited comparable performance to TD children regarding most tasks. Differences between groups were maintained only in multitasking complex tasks (the effect decreased to *r* = 0.28) and in commission errors, where no motivational impact was noted. Therefore, it seems that motivation, especially in simple cognitive tasks, can increase perceptual sensitivity in children with ADHD, increase the quality of information obtained from a stimulus, as well as the rate of collecting this information. However, motivation is not able to eliminate the limitations of complex tasks which require the integration of many cognitive processes, especially those more closely related to inhibitory control.

Our conclusions are supported by the Barkley model [72], which assumes that children with ADHD have limited capacity to self-regulate their affect and emotions and are more dependent on immediate and external sources of motivation. Children with ADHD can improve their performance under feedback control and from a reward perspective. Such relation was also observed by McInerney and Kerns [44] in the case of inhibitory control and working memory. In their study, the results of children with ADHD improved under the influence of motivation, but they still differed from those of the control group (children without disorders did not improve their performance under conditions of increased motivation). In the Reijnen and Opwis study [45] in conditions of increased motivation, children with ADHD exhibited comparable performance in visual search to those in the control group. Therefore, it seems that self-regulation of effort may be one of the key deficits in children with ADHD.

Our study is affected by certain limitations. It was conducted on a small number of participants. Further research is needed to generalize conclusions. In the study, we did not control the ADHD types of presentation or the co-occurrence of other disorders (e.g. behavioral disorders) that could differentiate the results. The structure of the group (age and sex) did not allow for assessing the demographic differences with respect to the controlled dependent variables. However, studies to date have indicated a statistically insignificant or small impact of age and sex on the TBR and HEG ratio values [73–75]. At the same time, researchers have observed significant improvement in the brain functioning between children and adults with ADHD, which may explain the weakening of attention deficit symptoms with age—especially with respect to hyperactivity (the inattentiveness component seems to be more stable) [76]. The HEG System allowed for assessing rCBO‌_2_ in the brain tissue at a depth of about 1.5 cm. The use of additional imaging techniques would allow for a better assessment of brain activity (e.g. SPECT). Two motivational techniques were applied in the study in parallel: the reward effect and the competition effect. We are not able to determine which of them proved to be more effective. Taking these variables into account may serve as an inspiration for further research. Despite the aforementioned limitations, our report introduced new data regarding cortical activity, motivation and attention deficits in children with ADHD.

## Summary

The study was conducted in the Fall of 2019 in Kraków (Poland). It included 30 children with ADHD as well as 30 TD children. The study confirmed significant differences between children with ADHD and TD children in terms of TBR and HEG ratio, which indicate different patterns of cortical activity in prefrontal and central areas. The study also confirmed the occurrence of attention deficits in children with ADHD. Moreover, for the first time we have displayed differences in motivation dimensions between ADHD and TD children as per the self-determination theory. We have also confirmed the positive impact of extrinsic motivation on cognitive capacity in children with ADHD—this is the first study to assess the impact of extrinsic motivation on vigilance and divided attention. The obtained data allow for a better understanding of the issues and for defining the clinical picture of ADHD. Cognitive deficits in children with ADHD are not permanent neuropsychological impairments. Cognitive capacity may be modulated by means of motivation. Evoking interest and more frequent strengthening help reduce cognitive deficits in children with ADHD. The data have an application value. They may be used in the diagnosis of ADHD, in the development of pedagogical programs and in therapeutic work.
